# Physical Activity in Hemodialysis Patients Measured by Triaxial Accelerometer

**DOI:** 10.1155/2015/645645

**Published:** 2015-05-24

**Authors:** Edimar Pedrosa Gomes, Maycon Moura Reboredo, Erich Vidal Carvalho, Daniel Rodrigues Teixeira, Laís Fernanda Caldi d'Ornellas Carvalho, Gilberto Francisco Ferreira Filho, Julio César Abreu de Oliveira, Helady Sanders-Pinheiro, Júlio Maria Fonseca Chebli, Rogério Baumgratz de Paula, Bruno do Valle Pinheiro

**Affiliations:** ^1^Pulmonology Division, Faculty of Medicine, Federal University of Juiz de Fora, 36036-110 Juiz de Fora, MG, Brazil; ^2^Internal Medicine Department, Faculty of Medicine, Federal University of Juiz de Fora, 36036-110 Juiz de Fora, MG, Brazil; ^3^Interdisciplinary Center for Studies, Research and Treatment in Nephrology (NIEPEN), Federal University of Juiz de Fora, 36036-110 Juiz de Fora, MG, Brazil

## Abstract

Different factors can contribute to a sedentary lifestyle among hemodialysis (HD) patients, including the period they spend on dialysis. The aim of this study was to evaluate characteristics of physical activities in daily life in this population by using an accurate triaxial accelerometer and to correlate these characteristics with physiological variables. Nineteen HD patients were evaluated using the DynaPort accelerometer and compared to nineteen control individuals, regarding the time spent in different activities and positions of daily life and the number of steps taken. HD patients were more sedentary than control individuals, spending less time walking or standing and spending more time lying down. The sedentary behavior was more pronounced on dialysis days. According to the number of steps taken per day, 47.4% of hemodialysis patients were classified as sedentary against 10.5% in control group. Hemoglobin level, lower extremity muscle strength, and physical functioning of SF-36 questionnaire correlated significantly with the walking time and active time. Looking accurately at the patterns of activity in daily life, HDs patients are more sedentary, especially on dialysis days. These patients should be motivated to enhance the physical activity.

## 1. Introduction

Sedentary lifestyle is considered a major risk factor for global mortality, especially for cardiovascular diseases, and to reduce this risk at least 30 minutes of daily moderate-intensity aerobic physical activity on five days each week is recommended [1]. Despite the well-known benefits of physical activity, available data suggest that nearly 31% of the world's population does not achieve the minimum recommended [2]. In hemodialysis (HD) patients, physical inactivity may be even worse, due to the frequent presence of different comorbidities, such as anemia, uremic neuro- and myopathy, bone and mineral disorders, cardiovascular abnormalities, and depression [3, 4]. As an additional factor, the 12-hour a week dialysis period may contribute to HD patients inactivity [5]. At the same time, the impact of the sedentary lifestyle on mortality tends to be higher among HD patients, as they have elevated cardiovascular risk and mortality 10 to 30 times higher than general population [6].

To our knowledge, the few studies that have assessed the physical activity level among HD patients demonstrated that they are more sedentary than healthy individuals [7, 8]. However, the assessments were made by questionnaires or activity-related energy expenditure measurements, which provide inaccurate data [9, 10]. Daily physical activity can be evaluated more precisely using triaxial accelerometers that quantify the time spent in different activities (e.g., walking) and body positions (e.g., standing, sitting, and lying down), as well as the number of steps taken. Accelerometers are considered the best monitor to assess common physical activities because they have greater accuracy and less variability compared to other measurement tools, and their results are comparable to video recordings, the “gold standard” [9, 10]. Determining the time in which HD patients are active during daily life allows us to identify those who are sedentary and may call attention of the caregivers to the problem, so they can educate the patients to change their behavior.

Accurate assessment of physical activity in daily life is also important to investigate its association with physiological, clinical, and laboratory parameters, which may aid in understanding the mechanisms involved in sedentary behavior and even in suggesting changes in it.

Therefore, our objective was to compare physical activity in daily life in HD patients with control individuals, by using a triaxial accelerometer, and to investigate variables associated with inactivity.

## 2. Methods

### 2.1. Study Design

We conducted a single-center case-control study comparing HD patients from the Nephrology Unit of the University Hospital of the Federal University of Juiz de Fora, Juiz de Fora, Brazil, with control individuals. We recruited and evaluated the patients consecutively between March 2012 and April 2013.

### 2.2. Patients

Nineteen patients were recruited from outpatient dialysis facilities. Eligibility criteria included age ≥18 and <65 years and HD three times per week (Monday, Wednesday, and Friday), totaling 12 hours weekly, for at least 6 months. Exclusion criteria were uncontrolled arrhythmia, unstable angina, uncontrolled hypertension with systolic blood pressure ≥200 mmHg and/or diastolic blood pressure ≥110 mmHg, uncontrolled diabetes mellitus, severe respiratory diseases, acute infection, severe renal osteodystrophy, and neurologic or musculoskeletal disturbances.

The control group was comprised of subjects without chronic kidney disease, matched with the patients by age and gender, and recruited from the local community, among relatives of students and employees of the hospital.

None of the participants from both groups had been involved in any kind of exercise training in the preceding 6 months. The study protocol was approved by the Research Ethics Committee of the Federal University of Juiz de Fora, and all patients signed an informed consent form.

### 2.3. Measurements and Outcomes

#### 2.3.1. General Data

Demographic and clinical data, including age, gender, race, comorbidities (diabetes, hypertension, and cardiovascular disease), education level, family income, and body mass index (BMI), were collected by retrieving medical records and asking the patient directly.

#### 2.3.2. Physical Activities in Daily Life by Accelerometer

A triaxial accelerometer (DynaPort MiniMod; ©McRoberts BV, Netherlands) was used to assess physical activities in daily life. It consists of a small box, of 78 g (batteries included) and dimensions of 64 × 62 × 13 mm, which was enclosed in a belt worn on the waist. The device measures the time spent in standing, walking, sitting, and lying, and the number of steps taken. The accelerometer was used for 12 h/day, starting at awaking, for four consecutive weekdays, which corresponded, in HD patients, to two dialysis days and two nondialysis days. The first day of analysis was always the day after the first dialysis session of the week. The mean of the values obtained for each day was used for the statistical analysis. The monitor accuracy and technical specifications can be found elsewhere [11].

Patients were classified as sedentary, somewhat active, or active, based on the numbers of steps taken (<5000, 5000 to 7499, or ≥7500 steps/day, resp.) [12]. It was not possible to “blind” patients concerning the use of the device, as they were informed about the study before signing the consent form. However, both groups received the same information and all were instructed to maintain their usual activities of daily life.

#### 2.3.3. Six-Minute Walk Test (6MWT)

The analysis of physical functioning was performed by 6MWT during the nondialysis day, following the recommendations of the American Thoracic Society [13]. Patients were instructed to walk as fast as possible during the 6 minutes on a flat 30-meter track, and the distance walked was recorded in meters. Patients were allowed to stop and rest during the test but were instructed to resume walking as soon as they felt able to do so. Two tests were completed on the same day, with an interval of 30 minutes between each, recording the greater distance obtained.

#### 2.3.4. Peripheral Muscle Strength

Isometric handgrip strength was measured with a hydraulic hand dynamometer (Jamar Preston, Jackson, MI, USA). Subjects were instructed to self-adjust the dynamometer, with the elbow at 90° of flexion, forearm, and wrist in 3 neutral positions, and to grip the dynamometer with maximum strength after voice command. At least 3 attempts were performed, with a rest period of at least 1 minute between trials and the highest value was used in the analyses and related to the reference values [14]. In HD patients, the measurements were made at the nonfistula side during nondialysis day, and in control subjects they were made at the dominant side.

The sit-to-stand test was used to assess lower extremity muscle strength. Patients were instructed to stand up from a seated position and sit back down, with their arms folded across the chest, on a standard 44 cm straight-back chair with no arm rests. The number of repetitions achieved at the end of 60 seconds was recorded [15].

#### 2.3.5. Quality of Life Questionnaire (SF-36)

The SF-36 was used for the evaluation of quality of life [16]. The SF-36 is composed of 36 items that evaluate eight dimensions. In this study, the following dimensions were evaluated: physical functioning, physical role, vitality, social functioning, emotional role, and mental health. For each dimension, a score is obtained, with values varying from 0 (highly compromised) to 100 (not compromised).

#### 2.3.6. Laboratory Data

Creatinine, hemoglobin, albumin, serum iron, ferritin, calcium, phosphate, and dialysis adequacy (*Kt*/*V*) [17] were measured before the first HD session of the week.

### 2.4. Statistical Analysis

Statistical analyses were performed using SPSS version 17.0 for Windows (SPSS Inc., Chicago, IL, USA). Normal distribution was checked with the Kolmogorov-Smirnov test. Continuous variables were expressed as mean ± SD or median (interquartile range) according to whether they were normally distributed or not, respectively. Comparisons between groups were performed by unpaired *t*-test for normally distributed data or Mann-Whitney test for nonnormally distributed data. Dichotomous variables were expressed as percentages and comparisons were performed by chi-square test. Pearson's or Spearman's coefficient was used for the single correlation between the accelerometer outcomes with physiologic variables, for normally and nonnormally variables, respectively. The level of significance was set at *P* < 0.05.

## 3. Results

A total of 40 HD patients were initially assessed for eligibility. Of them, 12 were excluded, 9 declined to participate, and 19 entered the study. Characteristics of the HD patients and control subjects are shown in Table 1. Average age, gender distribution, and race were similar between the groups. Educational level, family income, and BMI were higher in the control group. As expected, the levels of creatinine, hemoglobin, albumin, serum iron, ferritin, and phosphate as well as the prevalence of comorbidities showed significant differences between HD patients and control subjects. The mean *Kt*/*V* measured in HD patients was 1.5 (±0.2), showing higher dialysis adequacy, and all of them were receiving erythropoietin.

**Figure 1:**
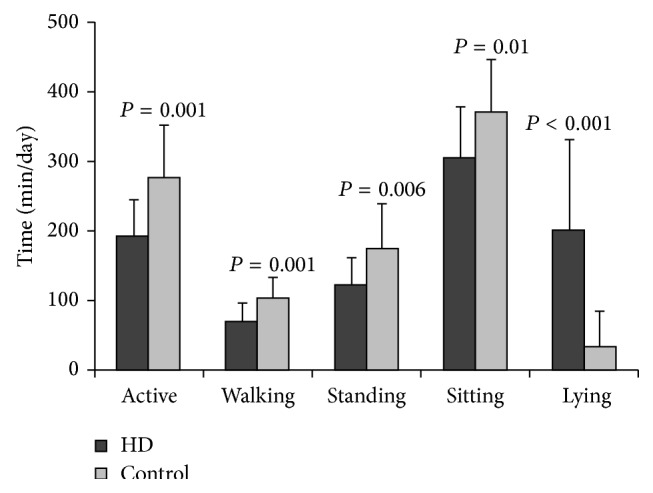
Time spent per day (minutes) in different activities in hemodialysis (HD) patients and control subjects, measured by triaxial accelerometer for 12 hours.

Figure 1 shows the time spent per day in different activities in both groups. The HD patients demonstrated a shorter activity time per day than control subjects (193.0 ± 54.0* versus *278.0 ± 76.1 min/day; *P* = 0.001), with shorter walking (70.1 ± 27.3* versus *103.2 ± 31.5 min/day; *P* = 0.001) and standing time (122.8 ± 40.4* versus *174.8 ± 65.0 min/day; *P* = 0.006). HD patients spent more time lying down per day than control subjects (202.2 (130.9)* versus* 32.7 (53.2) min/day; *P* < 0.001).

According to the numbers of steps taken per day, 47.4% of HD patients were classified as sedentary, whereas among control subjects this proportion was 10.5%. Also, 73.7% of control subjects achieved the minimum level of daily activity recommended, whereas only 21.0% of HD patients did. Furthermore, HD patients took fewer steps per day as compared to control subjects (5684 ± 2238 versus 8792 ± 2870 steps/day, *P* = 0.001).

Among HD patients, the sedentary behavior was greater on dialysis days, when they presented less active time, with shorter walking and standing time, and remained lying down for longer (*P* < 0.05). On dialysis days, the number of steps taken was significantly smaller than on nondialysis days (Table 2).

Compared to control subjects, HD patients were markedly more sedentary on dialysis days, with lower active, walking, and standing time, longer lying down time, and lower number of steps taken (*P* < 0.05). However, on nondialysis days, only the time spent lying down was significantly greater in HD group compared to control (Table 2).

Both groups presented normal pulmonary function and similar measurements of 6MWT, peripheral muscle strength, and the SF-36 domains physical role and vitality. The SF-36 domain physical functioning was significantly shorter among HD patients than control subjects (Table 3).

The hemoglobin level and physical functioning of SF-36 questionnaire correlated significantly with the walking time (*R* = 0.54, *P* = 0.003 and *R* = 0.43, *P* = 0.007) and active time (*R* = 0.41, *P* = 0.02 and *R* = 0.44, *P* = 0.006). The lower extremity muscle strength correlated significantly with active time (*R* = 0.40, *P* = 0.02).

## 4. Discussion

This study clearly shows that HD patients were more sedentary than the control subjects: they spent less time walking or standing and took fewer steps per day. Among them, the level of physical activity was lower on dialysis days, in which they were particularly more sedentary to the controls. On days without dialysis, HD patients showed behavior similar than control subjects, but they still remained lying down longer. We also observed that the hemoglobin level and physical functioning of SF-36 were positively correlated with the walking time and active time. Furthermore, the lower extremity muscle strength correlated significantly with active time.

The finding of lower level of physical activity in daily life among HD patients was not a surprise. Other authors have found this sedentary behavior using questionnaires or equipment that assesses the number of steps per day, describe the level of physical activity in arbitrary units, or estimate the energy expended [5, 7, 8, 18, 19]. Although questionnaires have been used to evaluate physical activity in daily living, they are not accurate and have a large variability across different factors, such as age, educational level, and cognitive capacity [9]. To our knowledge, this is the first study that assessed in detail the time these patients spend in different activities of daily life (walking, standing, sitting, or lying). The HD patients walked for 30.4% less time, took 33.8% fewer steps, and spent 28.3% less time standing than controls. Another feature that shows the sedentary behavior in HD patients was the fact that they stayed 27.4% of the observation period lying down in comparison to 6.8% in control individuals.

There is strong evidence showing that a sedentary lifestyle is associated with a higher mortality risk, mainly in patients with high cardiovascular risk, such as HD patients [4, 20]. Therefore, this accurate measurement of physical activity in daily life in this population is important and can add valuable information regarding this subject. The accuracy of DynaPort has been validated in different populations. It is particularly more precise than the other options in detecting movements of lower intensity, which is useful in the study of less active populations, such as HD patients [21–23]. Furthermore, this accelerometer stores data continuously over long period and does not interfere with the daily routine of the individual.

In an effort to combat the sedentary lifestyle, different health organizations suggest levels of physical activity that should be maintained during daily life, most of them based on the time of aerobic physical activity or on the number of steps taken [1, 24]. The WHO recommends that adults between 18 and 64 years should do at least 150 minutes of moderate-intensity aerobic physical activity (e.g., walking) or at least 75 minutes of vigorous-intensity aerobic physical activity (e.g., running) throughout the week or an equivalent combination of moderate- and vigorous-intensity activity. All these activities should be performed in bouts of at least 10-minute duration [25]. Although patients in both groups have walked more than 30 minutes per day, therefore reaching more than 150 minutes per week, they did not necessarily achieve this level with continuous periods of at least 10 minutes, and they cannot be classified as active or not by this criterion.

Regarding the assessment of the level of physical activity based on the number of daily steps, recent studies classify people as sedentary if they take <5000 steps per day, low active if they take 5000 to 7500 steps per day, and active if they take >7500 steps per day. However, they recognize more benefits when 10000 steps/day, or even 12500 steps/day, are achieved [12]. Considering this limit of 7500 steps/day as a cutoff, only 21% of HD patients met the recommendations, compared with 73% in the control group. As well, 47% of HD patients were classified as sedentary, against 10% among the controls. This sedentary behavior among HD patients is associated with several unfavorable clinical outcomes, including increased risk of death from cardiovascular disease, and should be addressed by treatment strategies [20]. Programs of aerobic training during HD and ambulatory physical activity incentives are alternatives that have been proven effective in improving clinical outcomes in these patients [26–30].

Among the factors that contribute to physical inactivity among HD patients, the dialysis procedure itself is one of the most important. On days with dialysis, patients took fewer steps, walked and remained standing for a shorter time, and spent more time lying down, primarily because of the period of inactivity during the dialysis. Other authors have shown that these patients are 24% less active on dialysis days, confirming that the procedure is primarily responsible for the inactivity on these days [5]. On the other hand, our results showed that on nondialysis days, patients did not take significantly fewer steps or spend less time walking or standing, compared to the control group, although they spent more time lying down. We believe that subject's better clinical condition on nondialysis days and the fact that they are the days with more time available result in a greater concentration of the daily tasks done on them.

To identify possible factors associated with lower physical activity level among HD patients, other than the period of dialysis itself, we evaluated lung function, exercise capacity, muscle strength, biochemistry, and quality of life. Among them, the hemoglobin level was positively correlated with the walking time and active time, even though its mean level was 10.9 g/dL, with all patients receiving erythropoietin. This result was expected, since anemia is one of the factors associated with a sedentary lifestyle in different conditions. It reinforces the importance of treating anemia in chronic kidney disease [3]. In addition, the physical functioning of SF-36 questionnaire was correlated with the walking time and active time. This correlation shows that self-reported physical functioning was associated with physical activity accessed objectively by triaxial accelerometer. Similarly, in a cohort study of patients starting dialysis, Johansen et al. show that the self-reported physical functioning was associated with quality of life [27]. In other studies, exercise capacity, peripheral muscle strength, and albumin have been associated with physical inactivity [8]. In our study, the lower extremity muscle strength evaluated by sit-to-stand test had a positive correlation with active time. Although the HD patients presented lower exercise capacity assessed by the 6MWT and lower levels of albumin, these variables did not correlate significantly with the level of physical activity, perhaps because of the low number of individuals studied.

A limitation of this study was the small number of patients enrolled. Although it has been sufficient to demonstrate the difference in the level of physical activity in daily life between the two groups, it may have compromised the analysis of the correlation of this with the variables studied. Also in relation to the inclusion of patients, it was not possible to match them regarding education level and family income. However, these differences probably did not compromise the results, because both variables, which usually have an inverse correlation with physical activity, were lower in the HD patients, making the sedentary lifestyles found in these patients even more significant. Another limitation could be the number of days evaluated, notably among HD patients. As the behavior is different between days with and without dialysis, it may be considered that just two days were evaluated in each condition. However, in patients with low levels of physical activity, the variability between days is small, and two days of monitoring seem to be sufficient [22]. Finally, DynaPort is expensive equipment when compared to other devices that assess physical activity, a fact that can limit its use on a large scale.

## 5. Conclusion

In conclusion, HD patients are less active than individuals without chronic kidney disease, especially on dialysis days. These patients should be motivated to enhance the physical activity.

## Figures and Tables

**Table 1:** Characteristics of patients on hemodialysis (HD) and control subjects.

Characteristics	HD patients (*n* = 19)	Control subjects (*n* = 19)	*P *
Age (years)	47.5 ± 12.5	46.7 ± 13.1	0.85
Male (%)	11 (58%)	12 (63%)	0.74
Race, nonwhite (%)	12 (57.9%)	6 (31.6%)	0.15
Comorbidities			0.001
Hypertension (%)	13 (68.4%)	2 (10.5%)	
Diabetes (%)	2 (10.5%)	1 (5.3%)	
Cardiovascular diseases (%)	4 (21.1%)	0 (0%)	
Educational level			0.01
≤4 years (%)	3 (15.8%)	0 (0%)	
>4 and ≤8 years (%)	7 (36.8%)	1 (5.3%)	
>8 years (%)	9 (47.4%)	18 (94.7%)	
Family income (USD/month)	580 (838)	2232 (1139)	0.001
BMI (kg/m^2^)	23.1 ± 3.9	26.1 ± 4.1	0.029
Creatinine (mg/dL)	12.2 ± 5.4	0.8 ± 0.2	<0.001
Hemoglobin (mg/dL)	10.5 (3.5)	13.8 (1.4)	<0.001
Albumin (g/dL)	3.8 (0.4)	4.0 (0.3)	0.03
Serum iron (*μ*/dL)	66.7 ± 25.9	125.6 ± 37.2	<0.001
Ferritin (ng/mL)	524.2 (418.3)	70.0 (114.6)	<0.001
Calcium (mg/dL)	9.5 ± 0.7	9.6 ± 0.5	0.58
Phosphate (mg/dL)	5.6 (2.8)	3.8 (0.9)	0.001
*Kt*/*V*	1.5 ± 0.2		

Data are mean ± SD or median (interquartile range) for symmetrically and asymmetrically distributed data, respectively. BMI: body mass index and *Kt*/*V*: dialysis adequacy.

**Table 2:** Characteristics of physical activities in daily life in hemodialysis (HD) patients on dialysis and nondialysis days and in control subjects.

	HD patients (*n* = 19)		Control subjects (*n* = 19)
	Dialysis days	Nondialysis days	
Active time (min)	142.9 ± 63.3^*^	242.9 ± 73.0	278.0 ± 76.1^#^
Walking time (min)	55.7 ± 30.8^*^	84.5 ± 38.1	103.2 ± 31.5^#^
Standing time (min)	87.2 ± 36.6^*^	158.3 ± 59.6	174.8 ± 65.0^#^
Sitting time (min)	311.2 ± 76.9	301.6 ± 88.6	372.2 ± 77.1^∗#^
Lying time (min)	250.1 ± 91.1^*^	152.1 ± 130.6	47.8 ± 70.5^∗#^
Steps/day	4362 ± 2084^*^	7007 ± 3437	8792 ± 2870^#^

^*^ *P* < 0.05 compared to nondialysis days; ^#^ *P* < 0.05 compared to dialysis days.

**Table 3:** Characteristics of pulmonary function test, six-minute walk test, peripheral muscle force, and SF-36 domains, in hemodialysis (HD) patients and control subjects.

	HD patients (*n* = 19)	Control subjects (*n* = 19)	*P*
6MWT, m	519.9 ± 94.5	569.1 ± 79.9	0.14
Isometric handgrip force, kgf	34.0 ± 9.4	39.2 ± 12.6	0.21
Sit-to-stand test, events per minute	26.0 (7.0)	29.0 (7.7)	0.33
*SF-36 domains *			
Physical functioning	74.2 ± 21.9	88.4 ± 13.5	0.02
Physical role	65.7 ± 44.2	82.6 ± 37.2	0.43
Vitality	71.3 ± 21.1	71.8 ± 25.2	0.91
Social functioning	90.7 ± 25.6	86.6 ± 21.3	0.07
Emotional role	77.1 ± 38.5	76.9 ± 40.0	0.48
Mental health	76.4 ± 18.8	83.3 ± 14.2	0.31

FEV_1_: forced expiratory volume in the first second; FVC: forced vital capacity; and 6MWT: 6-minute walking test.
